# Apixaban plasma concentrations in patients with obesity

**DOI:** 10.1007/s00228-024-03696-4

**Published:** 2024-06-01

**Authors:** Fadiea Al-Aieshy, Mika Skeppholm, Jonas Fyrestam, Fredrik Johansson, Anton Pohanka, Rickard E. Malmström

**Affiliations:** 1 Department of Medicine Solna, Karolinska Institutet & Clinical Pharmacology, Karolinska University Hospital Solna, 17176 Stockholm, Sweden; 2 Department of Clinical Sciences, Karolinska Institutet & Division of Cardiovascular Medicine, Danderyd Hospital, Stockholm, Sweden; 3 Department of Clinical Pharmacology, Karolinska University Hospital, Stockholm, Sweden; 4 Department of Clinical Sciences, Karolinska Institutet & Medical library, Danderyd Hospital, Stockholm, Sweden; 5 Department of Laboratory Medicine (LABMED), Karolinska Institutet & Clinical Pharmacology, Karolinska University Hospital, Stockholm, Sweden

**Keywords:** Obesity, Body weight, Apixaban, Plasma concentration, Therapeutic drug monitoring

## Abstract

**Purpose:**

Routine therapeutic drug monitoring of apixaban is currently not recommended but may however be warranted in some situations and for some patient groups to provide better and safer treatment. Due to limited data on apixaban concentrations in different subpopulations, it is still unclear which group of patients could possibly gain from monitoring. The purpose of this study was to examine apixaban exposure in patients with obesity compared with normal-weight patients.

**Methods:**

Forty patients with obesity (mean BMI 39.4 kg/m^2^) and 40 controls with normal weight (mean BMI 23.4 kg/m^2^), treated with apixaban 5 mg twice daily were included. The patients were matched for age, sex, and renal function. Trough and peak apixaban concentrations were measured with LC‒MS/MS methodology.

**Results:**

The median trough concentrations in patients with obesity (58.7, range 10.7–200.7 ng/ml) were slightly higher than those in patients with normal weight (52.0, range 31.0–150.9 ng/ml) (*p* < 0.05). Notably, the variability in trough concentration was considerably higher in patients with obesity. Peak concentrations were similar in both groups, with a median of 124.5 ng/ml (range 82.0–277.5) and 113.5 ng/ml (range 75.5–334.6) in patients with obesity and normal weight, respectively.

**Conclusion:**

Apixaban exposure did not vary substantially between obese and normal weight matched controls, implying that general dose adjustments are not required. However, vast interindividual variability was observed in patients with obesity, suggesting that measuring the concentrations could be valuable for specific patients. Further research is needed to identify which specific patients may benefit from this approach.

**Supplementary Information:**

The online version contains supplementary material available at 10.1007/s00228-024-03696-4.

## Introduction

Obesity and overweight are a growing global health problem with a prevalence that has almost tripled since the 1970s. In 2016, 39% (1.9 billion) of the adult population were overweight, around one-third of which were obese. A body mass index (BMI) ≥ 25 kg/m^2^ is classified as overweight, and ≥ 30 kg/m^2^ is classified as obese [1]. According to the World Health Organization (WHO), obesity and overweight are defined as “abnormal or excessive fat accumulation that presents a risk to health.” Obesity is estimated to be the primary cause of approximately four million deaths each year. With a higher BMI, the risk for several diseases increases, e.g., diabetes, cancer, and cardiovascular diseases, such as atrial fibrillation (AF), atrial flutter (AFL), and venous thromboembolism (VTE), including deep vein thrombosis (DVT) and pulmonary embolism (PE) [2–5]. For the prevention of stroke and systemic embolism in patients with AF and for the treatment/prevention of DVT and PE, oral anticoagulants are used: direct oral anticoagulants (DOACs) or warfarin [6].

The European Society of Cardiology (ESC) recommends using DOACs as first-line therapy instead of warfarin, and the direct factor Xa (FXa) inhibitor apixaban is currently on the top ten list of best-selling drugs globally [7–9]. One of the advantages of apixaban and other DOACs is that they, in contrast to warfarin, can be prescribed in a fixed-dose management without the need for routine laboratory monitoring. However, there is an exposure dependency in safety and efficacy, and although routine clinical monitoring is currently not recommended in general for the entire population, in some clinical situations and for some patients, monitoring apixaban concentration could be helpful [10–13]. Apixaban is a potent drug that can cause serious adverse bleeding if drug exposure is too high but also severe consequences if it is too low, as thrombosis might occur. ISTH SSC guidelines from 2016 suggest that apixaban should not be used in obese with BMI > 40 kg/m^2^ or weight > 120 kg, and if used, monitoring is recommended [14]. Updated guidelines for VTE were published in 2021 and recommended the use of apixaban in standard doses for the treatment or prevention of VTE in patients with high BMI/weight [15]. However, to our knowledge, this update was based on very limited new published clinical data on patients with obesity. Low body weight is included among the criteria for dose reduction of apixaban, as it may contribute to higher exposure of the drug. Less is known about the opposite, i.e., high body weight, and there are consequently no dose adjustment recommendations provided even though in theory it could mean lower exposure to the drug in this population or in certain individuals [16].

There are scarce data on high body weight in the pivotal clinical trials for apixaban; in some trials, safety/efficacy analyses on patients with high body weight were not presented (AVERROES, AMPLIFY EXT) [17, 18]. However, in patients with acute VTE and a weight ≥ 100 kg or BMI > 35 kg/m^2^, superior safety was seen for apixaban compared with enoxaparin/warfarin, but no statistically significant difference was seen for the efficacy (AMPLIFY) [19]. A pooled analysis of ADVANCE 2 and 3 showed no difference in either safety or efficacy for patients with BMI ≥ 30 kg/m^2^ treated with apixaban compared to enoxaparin in the prevention of VTE after orthopedic surgery [20–22]. Furthermore, a post hoc analysis of the ARISTOTLE trial (AF patients) showed equal safety and efficacy for apixaban compared with warfarin treatment in patients with weight > 120 kg [23].

In the pivotal studies, the efficacy and safety of apixaban in subpopulations of extreme body weight have thus been presented compared to other means of anticoagulation (i.e., conventional therapy). There is very little knowledge regarding how and whether treatment with apixaban can be optimized at a subpopulation or individual level, as there are limited data on measurements of apixaban plasma concentrations in different sets of patients. The aim of this study was to assess apixaban exposure in patients with obesity compared with normal weight patients with AF/AFL, DVT, or PE.

## Methods

This non-interventional prospective study was performed between January 2020 and November 2022. Patients treated with apixaban 5 mg twice daily due to AF/AFL, DVT, or PE according to current clinical recommendations and the summary of product characteristics for Eliquis were recruited from the cardiology department at Danderyd Hospital. Patients with the following criteria were excluded: relative estimated glomerular filtration rate (eGFR) < 30 ml/min/1.73 m^2^, bariatric surgery, concomitant treatment with strong/moderate CYP3A4 and P-gP inhibitors based on the recommendations in Stockley’s Drug Interactions database and Janusmed drug interaction database (previous name SFINX) [24–26]. For the calculation of relative eGFR, the revised Lund-Malmö formula (LM-rev) was used [27]. Creatinine, sex, and age were used to generate the body surface-adjusted relative eGFR. In addition to relative eGFR, absolute eGFR was also calculated using patient length and total body weight (TBW) or ideal body weight (IBW) for estimation of body surface area using the DuBois formula [28]. The IBW was calculated according to the Devine formula (1974) for women as 45.5+(0.91*(length−152.4)) and men 50+(0.91*(length−152.4)) [29].

Patients with a BMI > 30 kg/m^2^ were enrolled in the obese group and 18.5–24.9 kg/m^2^ in the normal weight group. Information regarding BMI and weight was extracted from the patients’ medical records or obtained directly from the patients themselves via telephone call. A patient visit was booked during which blood sampling and various measurements were performed at steady state after at least 3 days of apixaban treatment of 5 mg twice daily. Blood samples were collected for analysis of apixaban concentration and s-creatinine. Further activated partial thromboplastin time (aPTT), prothrombin time-international normalized ratio (PT-INR), and hemoglobin (Hb) were analyzed (description of the methods, see Supplementary material). The actual weight, length, and waist size were measured during this patient visit. For each patient, age, sex, concomitant drug treatment, diagnoses, and smoking habits were registered. The information was obtained from the patient medical records and during meeting/contact with the patient.

A total of 40 patients with obesity and 40 normal weight patients, matched for age (± 6 years), sex, and relative eGFR (± 15 if > 60 mL/min/1.73 m^2^ and ± 10 if < 59 mL/min/1.73 m^2^), were included.

### Measurement of apixaban concentration

Blood samples were drawn by direct venipuncture from an antecubital vein and collected into vacuum tubes containing citrate (5/4.5). The blood samples were centrifuged (2000 ⋅ g) at room temperature for 20 min. Plasma was then aliquoted in 250 µl cryotubes and frozen at −80 °C. The frozen samples were sent in a batch to the Clinical Pharmacology unit at the Karolinska University Hospital for measurement of apixaban concentrations using a gold standard LC‒MS/MS method modified from a previously described method [30]. The method is described in Supplementary material.

### Statistical analysis

To estimate the relationship between different variables, Pearson’s correlation analysis was used. In addition, a multiple regression analysis was performed. Differences between groups of patients (e.g., obese and normal weight) in apixaban plasma concentrations were evaluated by Wilcoxon signed rank test for related samples. To estimate differences in patient characteristics between the obese and matched normal weight control patients, a paired sample *t* test was used for normally distributed variables, and for non-normal variables, the Wilcoxon signed rank test was used. For nominal variables, e.g., comorbidities, McNemar’s test was used. Normality distribution was assessed with histograms and the Shapiro‒Wilk or Kolmogorov‒Smirnov tests.

With 40 patients in each group, we have a 90% power to detect a difference of 25 ng/mL between the matched groups with a standard deviation of 31, a significance level of 5%, and a paired *t*-test, accounting for a 20% dropout rate.

A *p* value < 0.05 was considered statistically significant. Statistical analysis was performed in IBM SPSS version 25 (IBM Corp, USA).

## Results

### Cohort characteristics

A total of 80 patients treated with apixaban 5 mg twice daily were included in this paired analysis, forming 40 matched obese and normal weight pairs. A higher percentage of males were included (72.5%, 29 pairs). VTE was the predominant therapeutic indication for apixaban therapy in 61 patients (76%), while 17 patients were prescribed apixaban for AF/AFL (21%) and two patients for a combination of AF/AFL/VTE (3%). The proportion of comorbidities such as diabetes, hypertension, and/or heart failure was higher in patients with obesity with 65% (20% diabetes, 63% hypertension, 10% heart-failure) compared to 35% (10% diabetes, 25% hypertension) in normal weight patients, *p* < 0.05. Patient characteristics are presented in Table 1. Weight, BMI, and waist size were the only characteristics that differed between the two groups. When using the TBW to calculate the absolute eGFR, the groups were significantly different. While this was expected due to the extreme body weight in the obese group, absolute eGFR IBW showed no difference between the two groups. There were no significant differences between the groups for aPTT, PT-INR, or Hb (data presented in the supplementary material, Table S1).

**Table 1:** Patient characteristics for the two paired groups: patients with obesity and normal weight patients

**Variable**	**Obese, *n* = 40**	**Normal weight, *n* = 40**	***p* value**
**Age (years)**			
Mean (min–max)	57.5 (32.0–81.0)	58.2 (28.0–79.0)	NS
Median (IQR)	56.0 (50.3–64.0)	58.5 (50.3–65.0)	
**Weight (kg)**			
Mean (min–max)	124.4 (90.6–183.1)	75.0 (57.1–93.4)	< 0.001
Median (IQR)	120.1 (108.8–136.1)	77.5 (69.0–80.8)	
**BMI (kg/m**^**2**^**)**			
Mean (min–max)	39.4 (31.7–51.5)	23.4 (18.7–26.1)	< 0.001
Median (IQR)	38.5 (36.4–42.4)	23.5 (22.3–24.4)	
**Waist size (cm)**			
Mean (min–max)	124.9 (98.0–167.0)	87.6 (71.0–101.0)	< 0.001
Median (IQR)	126.3 (114.4–134.4)	89.0 (84.0–92.0)	
**Length (cm)**			
Mean (min–max)	177.5 (148.0–200.5)	178.8 (161.0–202.5)	NS
Median (IQR)	181.3 (165.9–185.9)	179.2 (173.5–184.5)	
**Relative eGFR (ml/min/1.73 m**^**2**^**)**			
Mean (min–max)	76.9 (54.2–108.4)	77.1 (53.7–97.9)	NS
Median (IQR)	76.6 (68.4–85.7)	76.7 (72.1–83.5)	
**Absolute eGFR IBW (ml/min)**			
Mean (min–max)	83.7 (51.3–114.2)	85.1 (59.5–114.0)	NS
Median (IQR)	87.8 (73.4–94.7)	85.9 (75.1–95.1)	
**Absolute eGFR TBW (ml/min)**			
Mean (min–max)	105.9 (68.5–142.1)	86.1 (58.9–109.7)	< 0.001
Median (IQR)	107.0 (91.0–120.0)	86.5 (76.4–94.6)	

Data are presented as the mean (min–max) and median (interquartile range = IQR). A paired sample *t* test was used to calculate the *p* value for all variables except for weight, where the Wilcoxon signed rank test was used

### Apixaban trough concentrations

**Fig. 1:**
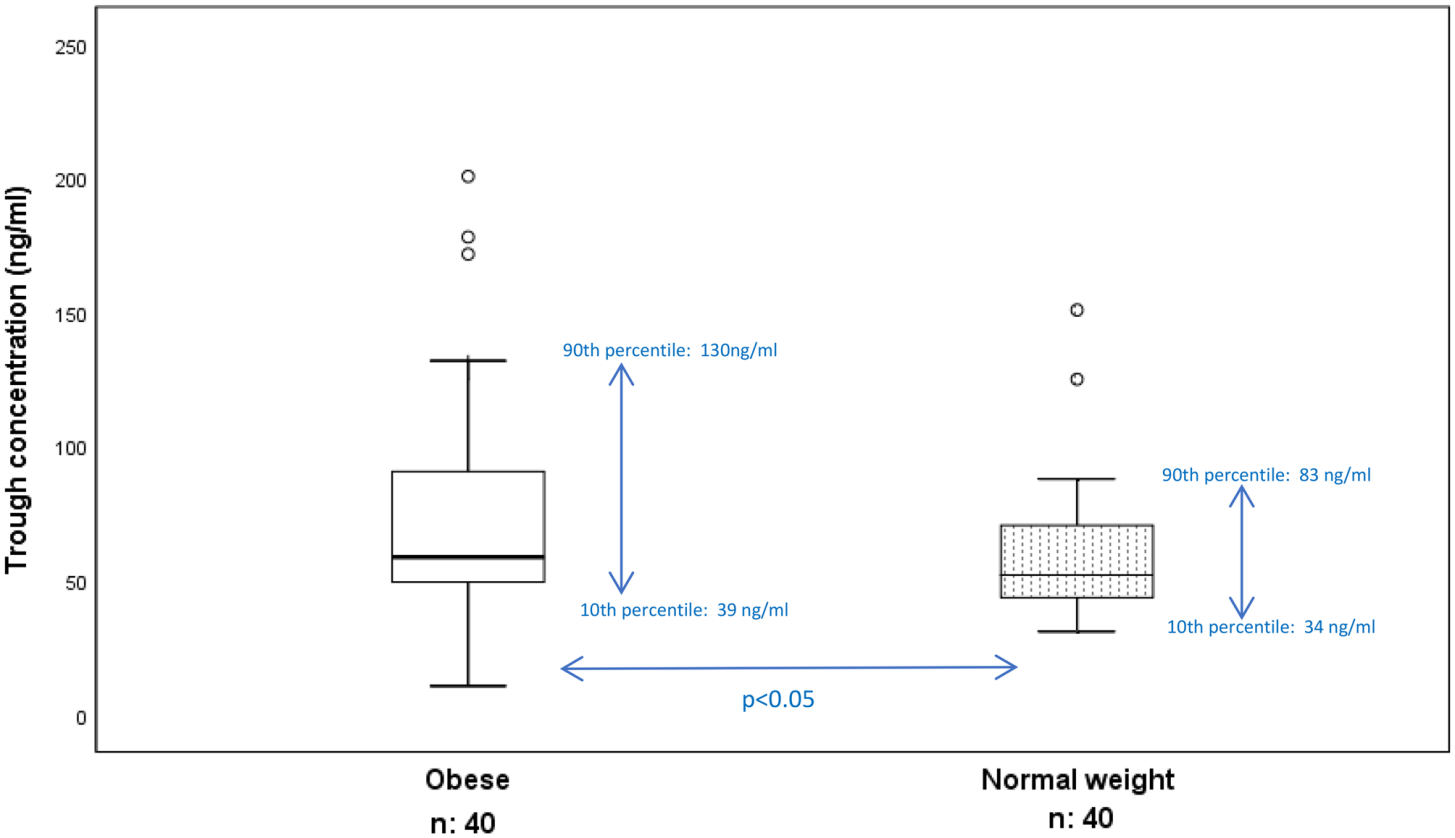
Apixaban trough concentrations measured in 40 patients with obesity and 40 matched normal weight patients treated with apixaban 5 mg twice daily. The line inside the box plot represents the median, and the top and bottom lines of the box show Q1 (25th percentile) and Q3 (75th percentile). The whiskers represent minimum and maximum values excluding outliers. Mild outliers (circle) are defined as values outside the ranges from 1.5 × interquartile range (IQR) below Q1 or above Q3

Blood samples for apixaban trough plasma concentrations were collected in patients with obesity and in patients with normal weight on average 12.6 h (range: 9.8–16.3) and 12.4 h (range: 9.4–14.8), respectively, after the last apixaban dose intake. Patients with obesity had slightly higher median trough apixaban concentrations than patients with normal weight, 58.7 (range 10.7–200.7) compared with 52.0 (31.0–150.9) ng/ml (*p* < 0.05). The coefficient of variation (CV) calculated as the ratio of the standard deviation to the mean was 56% for the patients with obesity and 42% for the normal weight patients. A more than 18-fold variation (calculated as the ratio between the highest and lowest value) in apixaban trough concentration was observed in the obese group, and the variation within the 10th–90th percentile was 3-fold (39.3–130.4 ng/ml) (Fig. 1). For the control group, the variation in trough concentrations was less pronounced, with a near 5-fold overall variation and a 2-fold variation within the 10th–90th percentile (34.0–83.1 ng/ml).

**Fig. 2:**
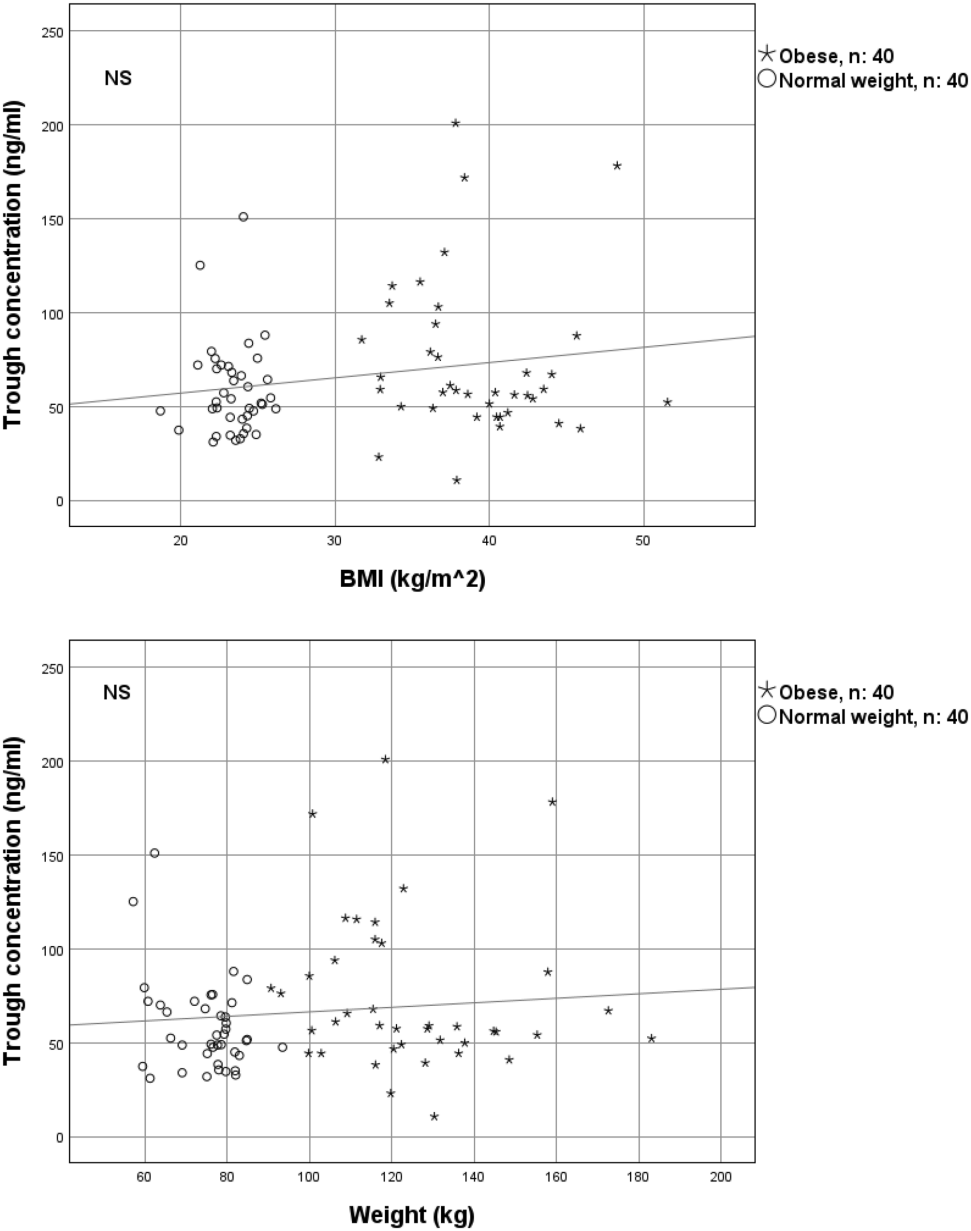
Correlation between apixaban trough concentrations and BMI/weight in 40 patients with obesity and 40 matched normal weight patients treated with apixaban 5 mg twice daily

There was no statistically significant correlation between trough concentrations and BMI or weight as visualized in Fig. 2. Among the 40 pairs, the trough concentrations were lower in patients with obesity than in normal weight patients in 12 pairs, and vice versa in 28 pairs.

### Apixaban peak concentrations

**Fig. 3:**
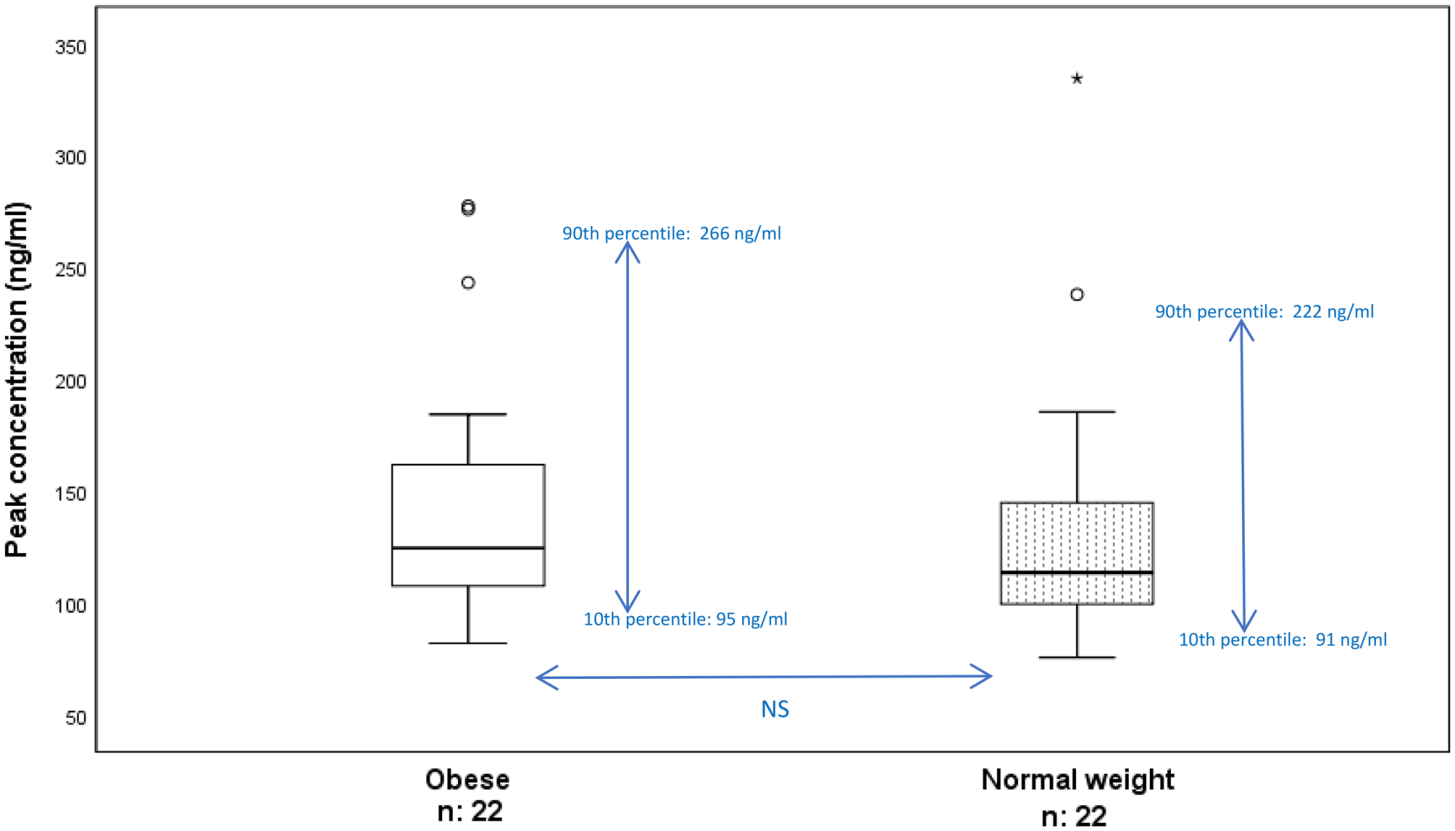
Apixaban peak concentrations measured with LC‒MS/MS methodology in 22 pairs of patients with obesity and normal weight patients treated with apixaban 5 mg twice daily. The line inside the box plot represents the median, and the top and bottom lines of the box show Q1 (25th percentile) and Q3 (75th percentile). The whiskers represent minimum and maximum values excluding outliers. Mild outliers are defined as values outside the ranges from 1.5 × interquartile range (IQR) below Q1 or above Q3. Extreme outliers (star) are defined as values outside the ranges from 3 × IQR below Q1 or above Q3

Peak sampling was collected from those who agreed on an additional blood sampling (meaning they had to stay another approximately 3 h for this), giving peak samples from 22 patients with obesity and 22 normal weight patients forming 22 pairs. The peak concentrations were collected on average 3.1 h (range 2.4–4.0) and 3.2 h (range 2.1–3.8) after the last apixaban dose intake in patients with obesity and normal weight patients, respectively. The obese had similar median peak concentrations as the normal weights (124.5, range 82.0–277.5 ng/ml, CV 39% in obese and 113.5, range 75.5–334.6 ng/ml, CV 43% in normal weights) (Fig. 3).

**Fig. 4:**
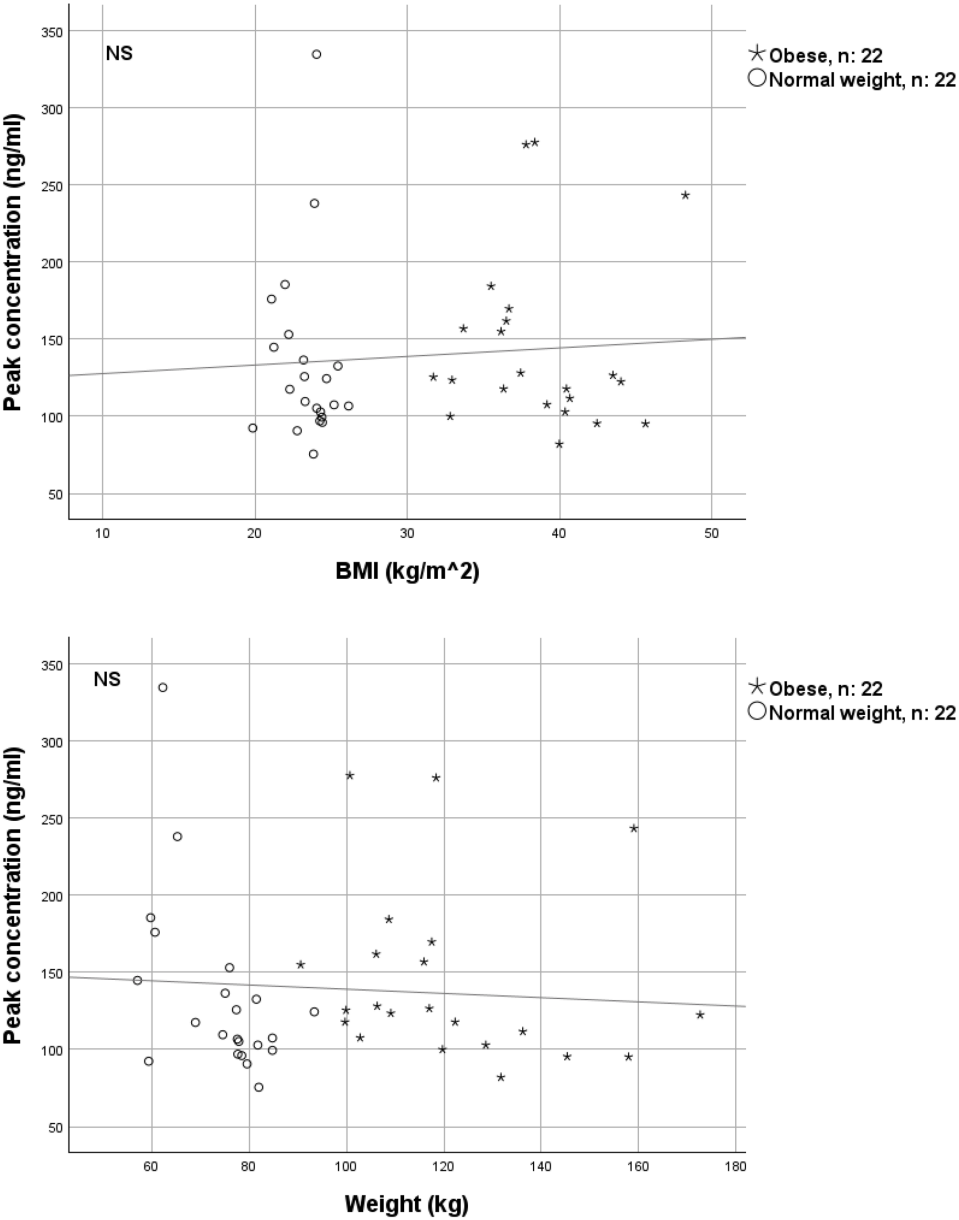
Correlation between apixaban peak concentrations and BMI/weight in 22 patients with obesity and 22 matched normal weight patients treated with apixaban 5 mg twice daily

**Fig. 5:**
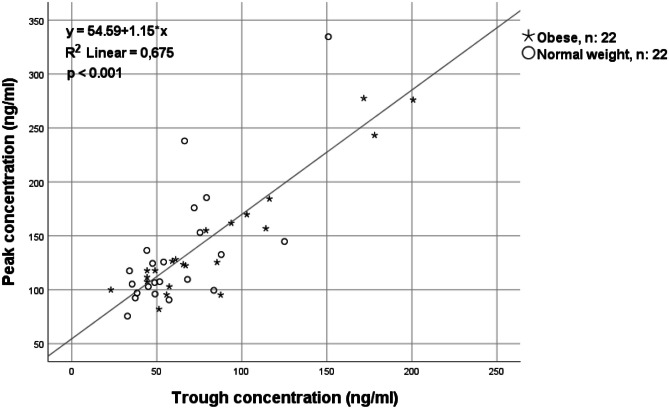
Correlation between apixaban concentrations at trough and peak in 22 patients with obesity and 22 matched normal weight patients treated with apixaban 5 mg twice daily

There were no significant correlations between peak concentrations and body weight/BMI (Fig. 4). A strong and significant correlation between trough and peak concentrations was observed; higher trough concentrations were correlated with higher peak concentrations (Fig. 5).

### Renal function

**Fig. 6:**
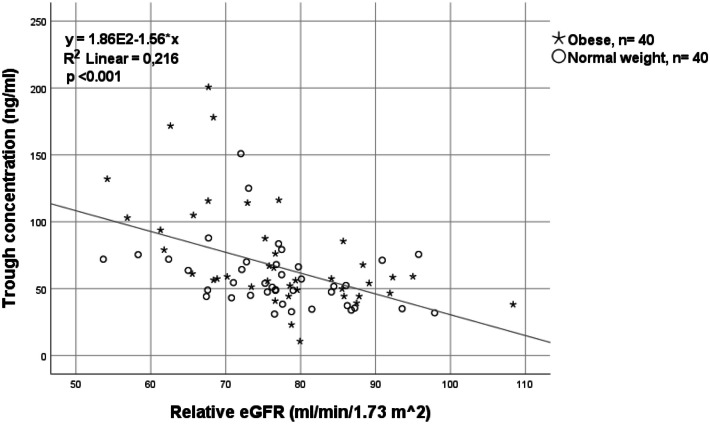
Correlation between apixaban trough concentrations and renal function estimated as relative eGFR in 40 patients with obesity and 40 matched normal weight patients treated with apixaban 5 mg twice daily

There were significant correlations between trough concentrations and renal function as measured by relative eGFR (Fig. 6) and absolute eGFR IBW (data not shown).

### Multiple regression analysis

The two groups (patients with obesity and normal weight patients) were individually matched for relative eGFR, age, and sex. Consequently, we conducted separate multiple regression analysis for each group. The multiple regression analysis was performed between trough concentration and body weight or BMI and relative eGFR and sex. For BMI and body weight, the results were non-significant. However, significant results were observed for relative eGFR for the analysis including BMI/body weight. For the BMI analysis, the following numbers were observed for relative eGFR: for the patients with obesity −2.12 (−3.16 to −1.08), *p* < 0.001 and for the normal weight patients −0.85 (−1.62 to −0.07), *p* = 0.033. Further, significant results were observed for patients’ sex but only for the patients with normal weight, and for the analysis including BMI, the numbers for female sex were 18.24 (0.84 to 35.64), *p* = 0.04).

### Outliers

Mild outliers are defined as values outside the ranges from 1.5 × interquartile range (IQR) below Q1 or above Q3. Extreme outliers (star) are defined as values outside the ranges from 3 × IQR below Q1 or above Q3. Six patients were defined as outliers at trough and/or peak, three obese and three normal weight. The three patients with obesity were defined as outliers at both trough and peak with trough concentrations between 171.7 and 200.7 ng/ml and peak concentrations between 243.2 and 277.5 ng/ml (Figs. 1 and 3). They were > 70 years old, had a BMI between 38 and 48 kg/m^2^, and two of the patients had a concurrent diagnosis of heart failure. Their relative eGFR was between 60 and 70 ml/min/1.73 m^2^ (Fig. 6). One normal weight patient was defined as an outlier at both trough and peak, at peak as an extreme outlier with a concentration of 334.6 ng/ml, and at trough a concentration of 150.9 ng/ml. In addition, one normal weight patient was defined as an outlier at peak (238.0 ng/ml) only and one patient at trough only (125.1 ng/ml) (Figs. 1 and 3). These three normal weight patients were between 45 and 80 years old, and none of them was diagnosed with heart failure. All outliers were included in the analyses.

### Subgroup analysis by patient’s sex

An exploratory subgroup analysis by patient’s sex was performed. Similar to the overall results, trough concentrations were, if anything, marginally higher in patients with obesity, both among female patients (median: 67.8 vs. 66.3 ng/ml, patients with obesity and normal weight respectively, *n* = 22 in total) and male patients (median: 57.4 vs. 49.1 ng/ml patients with obesity and normal weight respectively, *n* = 58 in total).

## Discussion

In our study of 40 obese-normal weighted matched pairs, weight or BMI did not correlate with either trough or peak concentrations. The patients with obesity had if anything slightly higher trough and peak concentrations, although for the peak concentrations, this was not significant. We found thus no evidence that patients with obesity in general are at risk of having lower exposure, i.e., concentration of apixaban in plasma, and therefore in need of higher doses of apixaban than patients of normal weight. Any differences that we found here between patients with obesity and normal weight patients were modest if any, and thus probably not of clinical relevance. The results from our study are consistent with other studies, in which apixaban concentrations were either decreased moderately or with no detected difference in apixaban exposure in obese subjects [31–34]. These studies were however either quite small and/or lacked a control group. The majority of the concentrations were within the reference range, which was determined based on apixaban concentrations measured in other studies. In one study [31], no relationship between apixaban concentrations and BMI or body weight was observed; however, in another study [34], whereas BMI did not correlate with trough or peak concentrations, weight had a moderate/weak negative correlation. In the abovementioned studies, the drug concentrations measured in obese were compared with apixaban concentrations from other studies. A strength of our study is that we compared the concentrations in obese with a control group constituted by matched normal-weight patients. The matched obese and normal-weight patients were thus similar for other known variables that could potentially affect the concentrations of apixaban. In contrast to the above, we found one study showing that healthy subjects with a body weight ≥ 120 kg and BMI ≥ 30 kg/m^2^ (*n* = 19) had 30% lower peak apixaban concentrations compared to normal weights (*n* = 16) [35]. This was, however, a single-dose study, and the results may not be transferable to patients receiving repeated doses.

The patients with obesity in our study exhibited a larger interindividual variation in trough concentration than the normal weight patients, more than 18-fold (range 11–201) compared with 5-fold (range 31–151). In our previous study in AF patients treated with apixaban 5 mg bid, a 6-fold variation was seen in trough concentration (range 29–186), again much lower variation than for the patients with obesity [30]. The observed larger variation in patients with obesity may indicate that this group has more unpredictable pharmacokinetics of apixaban. As exposure in obese seems less predictable, monitoring of apixaban concentrations could thus be considered. Especially if these patients have other factors that also may influence apixaban concentrations, e.g., medication with interacting drugs or renal insufficiency. An interesting observation from our study was that all three outliers in the obese group had relative eGFR below 70 ml/min/1.73 m^2^, and two of them had heart failure, indicating that these may be a subpopulation(s) within the obese at risk for very high apixaban exposure and where therapeutic drug monitoring is warranted. This is obviously based on very few observations and needs to be confirmed in a larger population. Another possible explanation for the large variability could be related to apixaban metabolism. Approximately 25% of apixaban is metabolized, mainly via CYP3A4 and to a lesser extent via other CYP450 enzymes [16]. Patients with obesity have been shown to have altered enzyme activity with a negative correlation between BMI and CYP3A4 activity. With weight loss, the activity of CYP3A4 was restored [36]. There are no data on apixaban exposure related to CYP3A4 activity in patients with obesity; thus, the clinical importance of this is uncertain. This is a field that is not fully studied, but one could speculate that the many physiological changes related to obesity may in turn relate to drug exposure variability. Obesity has been associated with different pharmacokinetic alterations not only for metabolism but also for drug absorption with accelerated gastric emptying and gastrointestinal transit, and for drug distribution with increased volume of distribution [37]. A population pharmacokinetic study of apixaban in AF patients showed that a body weight of 90 kg resulted in 22% increase in the volume of distribution compared to a 70-kg subject [38]. Furthermore, obesity is associated with other comorbidities that may give a higher variability between patients. In our study, the normal-weight patients were healthier, as the proportion of obese having other comorbidities was higher than the normal weight patients (65% compared with 35%). In addition, obese have a 3.5-fold increased risk of developing non-alcoholic fatty liver disease (NAFLD), which includes different liver conditions from liver steatosis to liver cirrhosis. In this study, we did not measure any liver function test other than PT-INR, which may be considered a limitation, as liver function impairment could impact apixaban metabolism and thus plasma concentrations [39].

## Conclusion

In summary, this study observed similar median apixaban plasma concentrations in patients with obesity and their sex-, age-, and renal function–matched controls with normal weight/BMI. However, the variability in plasma concentrations was considerably higher among the obese, and since it could be of importance to identify individuals with very high/low exposure, monitoring of apixaban plasma concentrations after initiation may have clinical relevance in this group.

### Electronic supplementary material

Below is the link to the electronic supplementary material.

Supplementary Material 1 (DOCX 30.3 KB)

## Data Availability

All available data are included in this published article (and its supplementary material).
