# Supplementary material for: Apixaban plasma concentrations in patients with obesity
Source: Eur J Clin Pharmacol. 2024 Jun 1;80(9):1343–54. doi: 10.1007/s00228-024-03696-4 (PMC11303434; doi:10.1007/s00228-024-03696-4)
Supplement: Supplementary file 1 — Supplementary Material 1 (DOCX 30.3 KB) [file 228_2024_3696_MOESM1_ESM.docx]

**Supplemental Information**

**Apixaban plasma concentrations in patients with obesity**

Fadiea Al-Aieshy^1^ 0009-0000-9977-4388, Mika Skeppholm^2^ 0000-0002-7746-3930, Jonas Fyrestam^3^ 0000-0002-5759-4861, Fredrik Johansson^4^, Anton Pohanka^5^ 0000-0001-9755-0778, Rickard E Malmström^1^ 0000-0003-3061-7629

*^1^Department of Medicine Solna, Karolinska Institutet & Clinical Pharmacology, Karolinska University Hospital, Stockholm, Sweden.*

*^2^ Department of Clinical Sciences, Karolinska Institutet & Division of Cardiovascular Medicine, Danderyd Hospital, Stockholm, Sweden*

*^3^Department of Clinical Pharmacology, Karolinska University Hospital, Stockholm, Sweden.*

*^4^Department of Clinical Sciences, Karolinska Institutet & Medical library, Danderyd Hospital, Stockholm, Sweden.*

*^5^Department of Laboratory Medicine (LABMED), Karolinska Institutet & Clinical Pharmacology, Karolinska University Hospital, Stockholm, Sweden.*

Corresponding author: Fadiea Al-Aieshy, [fadiea.al-aieshy@ki.se](mailto:fadiea.al-aieshy@ki.se)

Clinical Pharmacology, L2:04, Karolinska University hospital Solna, 17176 Stockholm, Sweden

**Laboratory methods**

Activated partial thromboplastin time (**aPTT**) and prothrombin time-international normalized ratio (**PT-INR)** were analysed on a Sysmex^®^ CS-5100 system, while hemoglobin (**Hb**) was analysed on a Sysmex XN-10/XN-20 (Sysmex, Kobe, Japan), all performed at the Department of Clinical Chemistry, Karolinska University Laboratory. The reagents used were Dade Actin FS APTT reagent (Siemens), MRX Owren´s PT reagent (Medirox) and sodium lauryl sulphate (SLS) for aPTT, PT-INR and Hb, respectively.

**Table S1** Laboratory values of aPTT, PT-INR and Hb for the two paired groups: patients with obesity and normal weight patients.

| Laboratory method | Obese, n=40 | Normal weight, n=40 | P value |
| --- | --- | --- | --- |
| aPTT (20-30 s)  - median (IQR) | 24.5 (23.3-26.0) | 24.5 (23.0-26.0) | NS |
| PK-INR (≤1.2)  - median (IQR) | 1.0 (1.0-1.1) | 1.0 (1.0-1.1) | NS |
| Hb  Female (117-153 g/L)  - median (IQR)  Male (134-170 g/L)  - median (IQR) | 136.0 (129.0-139.0)  149.0 (144.0-158.0) | 131.0 (123.0-137.0)  144.0 (138.5-152.0) | NS  NS |

Data are presented as the median (interquartile range = IQR). The Wilcoxon signed rank test was used to calculate the p value.

**Apixaban concentrations** were analysed using LC-MS/MS methodology, with a limit of quantification of 2 ng/ml. The calibration range for apixaban was 2-500 ng/mL and study samples were analysed together with quality controls (6 and 400 ng/mL) every twelve samples with defined acceptance criteria for batch release. Aliquots of 50 µL of blood plasma samples for standards, quality controls, blanks, and study samples, were precipitated with methanol containing 10 ng/mL of apixaban-^13^C_7_, used as an internal standard. Samples were vortexed for one minute, followed by centrifugation. Subsequently the supernatant was transferred and diluted with 0.1% formic acid, and vortexed for 10 seconds prior to analysis. For the analysis of apixaban an Acquity UHPLC I-Class PLUS system (Waters), connected to an Acquity UPLC BEH C_18_ 1.7 µm, 2.1 × 50 mm (Waters) column was used. The injection volume was 7 µL. Mobile phase A consisted of 0.1% aqueous formic acid (v/v) while mobile phase B comprised 100% methanol. Apixaban was eluted using a linear gradient program at a flow rate of 0.6 mL/min over a total runtime of 2.4 min, starting with 5% mobile phase B for 0.5 min followed by increase to 25% during 0.5 min, and held for additional 0.3 min. Mobile phase B was then increased to 30% over 0.7 min. and subsequently raised to 99% within 0.2 min, holding for an additional 0.1 min. Mobile phase B was then decreased back to its initial composition at 5%. The column temperature was set at 65 °C. For detection, a Xevo TQ-S micro (Waters) tandem quadrupole operated in positive electrospray ionization (ESI+) was employed. Selected reaction monitoring (SRM) with five SRM-scans for the same transition (*m/z* 460 > 443 for apixaban and *m/z* 468 > 451 for the internal standard) was employed to enhance the signal-to-noise ratio. The dwell time for each transition was automatically set to 0.011 s by the software, acquiring 12 data points over a 3 s-wide chromatographic peak. The capillary voltage was set to 0.8 kV, source temperature to 150 °C, desolvation temperature to 500 °C, cone gas flow to 0 L/hr, and desolvation gas flow to 1000 L/hr.
